# Contamination of microbial pathogens and their antimicrobial pattern in operating theatres of peri-urban eastern Uganda: a cross-sectional study

**DOI:** 10.1186/s12879-018-3374-4

**Published:** 2018-09-10

**Authors:** Sandra Matinyi, Muwanguzi Enoch, Deborah Akia, Valentine Byaruhanga, Edson Masereka, Isaac Ekeu, Collins Atuheire

**Affiliations:** 1 Department of Medical Laboratory Sciences, Faculty of Medicine, Mbarara of Science and Technology, (MUST), Mbarara, Uganda; 2 Department of Biosecurity, Ecosystems and Veterinary Public Health, College of Veterinary Medicine, Animal Resources & Biosecurity, Makerere University, Kampala, P.O Box 7076, Uganda; 3 Department of Public Health, School of Allied Health Sciences, Kampala International University, Western Campus, Bushenyi, Uganda

**Keywords:** Microbial pathogens, Operating theatre, Antimicrobial pattern

## Abstract

**Background:**

Microbial contamination of hospital environment, especially in operating theatres (OT) and other specialized units has greatly contributed to continuous and multiple exposure to nosocomial infections by patients and the public.

We purposed to assess microbial contamination of operating theatres and antibacterial sensitivity pattern of bacteria isolated from theatres of Mbale Regional Referral Hospital, Eastern Uganda.

**Methods:**

We employed a laboratory based cross-sectional study design. Swabbing of different surfaces and settle plate establishment in 4 various operating theatres was carried out. A total of 109 samples were collected, 31 air samples and 78 swabs from four operating theatres. Samples were collected in the mornings after disinfection prior to start of daily operations. Antibacterial sensitivity testing of isolated bacterial pathogens was performed by Kirby Bauer disc diffusion method following standard operating procedure. Colony counts for the settle plates were carried out using a colony counter.

**Results:**

All the four theatres had their mean colony counts exceeding the acceptable limit of 5 cfu/dm^2^/h. Gynaecology theatre had up to 261 cfu/dm^2^/h and Ophthalmology operating theatre had approximately 43 cfu/dm^2^/h. A total of 14 different organisms were isolated with *Pseudomonas* spp. [23.9%]; *Bacillus* spp. [17.5%] and *Aspergillus* spp. [15.8%] being the most common contaminants respectively. Other isolates included *Enterococcus* spp., *Rhizopus* spp. and Coagulate Negative *Staphylococcus* isolates especially from settle plates. Most bacterial isolates showed considerable resistance to antibacterial agents. *Pseudomonas* spp. was resistant to chloramphenicol (53.6%) and cotrimoxazole (57.1%). Most of the bacterial pathogens were sensitive to imipenem [83.3%].

**Conclusions:**

There is moderate contamination of operating theatres of Mbale Regional Referral Hospital. Common organisms were *Pseudomonas*, *Bacillus*, and *Aspergillus* spps. Resistance was observed against chloramphenicol and cotrimoxazole. More caution is necessary to carefully disinfect the operating theatres at Regional referral settings and similar tertiary health care centres with more emphasis on obstetrics and gynecology theatres. Diagnosis and care of patients at such clinical settings should consider the possibility of antibiotic resistance.

**Electronic supplementary material:**

The online version of this article (10.1186/s12879-018-3374-4) contains supplementary material, which is available to authorized users.

## Background

Microbial contamination of operating theatres has greatly contributed in precipitating the prevalence of hospital acquired infections, HAIs [1]. The burden of microbial contamination of operating theatres ranges from 4 to 37.4% pre and post operation [2–4], with an average of 11.5% pre-operation in Africa [5]. About 10% of these infections are responsible for adverse patient outcomes including increased mortality, increased length of stay of about 4–7 days among others [6]. In addition most of hospital acquired infections are drug resistant such as Methicillin resistant *Staphylococcus* aureus, *Pseudomonas* spp. [7, 8] and this complicates treatment. Anti-microbial resistance has become a big global public health problem arising from unregulated use of antimicrobials especially in developing countries [9].

The most implicated bacteria in operating theatre contamination include *staphylococcus species* accounting for about 40% [2, 3, 5] followed by Enterobacter and E. coli [5]. If the operating theatres are not sufficiently sterilized, surgical site infections are most likely to increase leading to poorer prognostic outcomes of patients post-surgery [10, 11] . Globally, surgical site infections (SSIs) account for up to 20% of all hospital associated infections [12].

Incidence of surgical site infection (SSI) in Uganda is 16.4% with Klebsiella spp. and *Staphylococcus aureus* being the most predominant; 50% and 27.8% respectively [13]. The study of microbial contamination in operating theatres is critical since patients who develop SSI are 60% more likely to spend more time in the hospital post-surgery [14].

Therefore clinical implication of bacterial contamination in operating theatre has enormous effects on both the patient as well as the caring medical team.

We purposed to assess microbial contamination of the operating theatre; [both surface and aerial space] as well as the antimicrobial pattern of the isolates at Mbale Regional Referral Hospital, Eastern Uganda.

## Methods

### Study design

A laboratory based cross-sectional study was carried out at Mbale Regional Referral Hospital between the months of August and October 2016.

### Study setting

Mbale Regional Referral Hospital (MRRH) is situated in Eastern part of Uganda in Mbale Town, a business centre in the region. The hospital currently serves 14 districts and provide both in-patient and out-patient services as well as laboratory services among others.

MRRH has got 460 bed capacity with an average annual attendance of 60,000 in-patients and 100,000 out-patients respectively. The hospital has got 5 operating theatres (OT); general private wing, Obstetrics & Gynaecology OT, Ophthalmology, Main general (General Public) and Casualty operating theatre. The later receives emergencies especially accident victims prior to General (Private or Public theatre).

### Sample size

The sample size (counting locations) was calculated according to the ISO 14644–2, which states that the numbers of counting locations are calculated according to the square root of the floor area (ISO, 2003) Table 1.

**Table 1:** Sample locations

Name of theatre	Square root of floor area	Locations
Obstetric surgery OT	3 M × 4 M = √12 ≈ 3	3
Ophthalmology OT	4 M × 5 M = √20 ≈ 4	4
Main OT general	4 M × 5 M = √20 ≈ 4	4
General private OT	3 M × 4 M = √12 ≈ 3	3
	Total	14

Since there was a chance of one microbe leaving one theatre to another, there was an underlying correlation that was dealt with by using a design effect of 2 (DE = 2.0). Hence, overall minimum sample size =14 × 2 = 28. Thirty-one locations for settle plates and 78 locations for swabs were identified. In each theatre, sampling was carried out in such way that the diagonals and the verticals were emphasized targeting the equipment in daily use, door handles as well as sinks. Settle plate locations were placed in areas adjacent to doors and in corners of operating theatres.

### Sample collection

The evaluation of microbial contamination in operating theatres was performed using Passive sampling method. Settle plate method was used for assessment of air and the swab method for assessment of surfaces. The samples were collected in the mornings after disinfection but before start of operations.

### Air sampling

Settle plates were used. Sterile open petri dishes of blood agar, chocolate agar, MacConkey and Sabouraud agar were labelled with a theatre identification number and placed in operating theatres to sediment air particles. The plates were each placed at the chosen places in the operation theatre at about 1 m above the ground, 1 m away from any obstacle and exposed for 1 h. After this exposure, the plates were each covered with its lid, placed in a zip lock bag and taken to microbiology laboratory.

### Surfaces sampling

SM and IE were involved in surface sampling. Sterile swabs were moistened in sterile saline and then, were used to swab the floor, walls, door handles, sinks, water taps, trolleys, operation tables and other equipment in the operating theatres. Some swabs were inoculated sterile peptone water and others in cooked meat medium. These swabs were labelled with theatre identification numbers and the spots from which they were taken and immediately transported to the microbiology laboratory for processing.

### Sample processing

SM, DA, VB and EM were actively involved in sample processing/laboratory analysis. In the laboratory, specimens in peptone water and cooked meat medium were respectively incubated aerobically and anaerobically at 37 °C overnight to resuscitate stressed organisms. Peptone water and cooked meat media specimens were then sub-cultured on blood agar, MacConkey, and Sabouraud agar. Settle plates and sub cultured plates except Sabouraud agar plates were incubated aerobically and anaerobically at 37 °C overnight. The plates were inspected for growth. When no growth was seen, the agar plates were re incubated for 24 h. When no growth was seen on both sub cultured agar plates and settle plates after re-incubation, they were discarded.

Sabouraud agar plates were incubated aerobically at 30 °C for up to 10 days while monitoring for growth. When no growth was seen, the sabouraud agar plates were discarded.

For plates that showed growth, microbial colonies were counted and the concentration of the airborne microorganisms were expressed as colony forming units per square decimeter per hour (cfu/dm^2^/h).

### Bacterial identification

The growth on culture plates was identified using conventional methods involving Gram’s staining technique, Lacto-phenol cotton blue and other appropriate biochemical tests. Including catalase, coagulase, oxidase, indole, motility tests, sugar fermentation tests, Triple sugar iron agar, citrate utilization and urease production. These were performed in accordance with the laboratory standard operating procedures adopted by Clinical and Laboratory Standard Institute (CLSI-M35-A2) [15]. Species characterization using ISO standards was not done due to logistical issues.

### Antimicrobial sensitivity testing

Antimicrobial susceptibility testing of isolated bacterial pathogens was performed by Kirby Bauer disc diffusion method. Inoculum was prepared by direct colony suspension method. Colonies were suspended in sterile saline, inoculum was then adjusted to turbidity equivalent to 0.5McFarland solution. Sterile swab was dipped into the suspension of the isolate in the sterile saline, squeezed free from excess fluid against the side of the bottle and spread over the Mueller-Hinton agar plate. Antibiotic discs appropriate for organisms were placed onto the media and incubated at 37 °C for 24 h. After 24 h each plate was examined and zones of inhibition were measured to the nearest millimeter, using sliding caliper which was held at the back of the inverted culture plate. The measurements were then compared with a standard chart as adopted by Clinical and Laboratory Standard Institute (CLSI) to determine susceptibility or resistance. The following agents were tested; chloramphenicol (30 μg), ampicillin (10 μg), amoxiclavin (30 μg), imipenem (10 μg), cotrimoxazole (25 μg), ciprofloxacin (5 μg), ceftriaxone (30 μg), gentamicin (10 μg) from BD, USA and tetracycline (30 μg) from Oxoid LTD, UK.

### Data management

Data was entered in Excel spread sheet, exported (Additional file 1: Data S1) to statistical package, STATA version 12 (Statacorp, College Station, 4905 Lakeway Drive, Texas 7784, USA) and Additional file 2: Data S2 exported to SPSS version 23 (IBM) for analysis. In both data sets data were fully described (Additional file 3). Summary of the common isolates was carried out using frequencies and proportions and was stratified across theatres. Comparison of findings across theatres as well as locations was carried out using Chi-square or Fisher’s exact test basing on expected frequencies. Graphical display of findings was carried out using bar graphs. Statistical significance of our findings was considered at *p* ≤ 0.05.

### Quality control

Settle plates were placed at the four corners of operating theatre room 15 min after realizing uniform air movement and instruments were swabbed diagonally to minimize sampling bias. The culture media used for isolation and identification of organisms (Blood Agar, DNase, Mannitol salt, Mac Conkey agar, Biochemical tests) were controlled using *S. aureus* ATCC 25923 and *E. coli* ATCC 25922 strains from WHO as per Laboratory Standard Operating Procedures.

Plate reading for isolation and identification of organisms was performed by two readers to minimize bias. Review of the results was done by a senior microbiologist at the laboratory to assure quality.

## Results

A total of 109 samples were collected. *n* = 31 were air samples and *n* = 78 were swabs. These were collected from four operating theatres (OT); Ophthalmology, General Private OT, Main General OT (Public), and Obstetric surgical OT.

### Bacterial load among the study operating theatres at Mbale regional referral hospital-eastern Uganda

Table 2 There was varied bacterial load (mean ± sd) among the operating theatres that were studied at Mbale referral hospital. Gynaecology theatre had the highest aerobic bacterial load, 261.3 ± 131.3 cfu/dm^2^/h followed by Main OT (general-public) with 69.5 ± 78.7 cfu/dm^2^/h. Ophthalmology theatre had the least numbers of bacterial load of approximately 44 cfu/dm^2^/h. However, the mean differences were not statistically significant between the theatres (F = 1.05, *p* = 0.38). Casualty OT was not sampled as this theatre operates all night and day and control of air flow is impossible. It receives accident victims [accidents often occur at night and early morning hours in Mbale Town]. Our criteria for sampling targeted morning hours before start of theatre operations and this precluded Casualty OT.

**Table 2:** Total aerobic bacterial load of air from the different operating theatres of Mbale Regional Referral Hospital, Eastern Uganda

Theatre	Mean cfu/dm^2^/h (sd)	F-test statistic	*p*-value
Gynaecology	261.3(131.3)	1.05	0.38
General-public	69.5(78.7)		
General-private	58.9(56.1)		
Ophthalmology	43.5(40.3)		

### Microbial isolates obtained from operating theatres of Mbale regional referral hospital-eastern Uganda

Table 3 Both gram-negative and positive bacteria were isolated in addition to fungi. *Pseudomonas* spp. were the most frequent (24%) followed by *Bacillus* spp. accounting for 17.5%. *Enterococcus*, *Rhizopus* ssp. were the least isolated organisms as low as < 0.5%.

**Table 3:** Frequency of microbial organisms in the operating theatres of Mbale Regional Referral hospital, Eastern Uganda

Organism	Frequency	Percent
*Pseudomonas* spp	56	23.9
*Bacillus* spp	41	17.5
*CoNS*	24	10.3
*Enterobacter aerogens*	20	8.5
*S. aureus*	18	7.7
*E.coli*	12	5.1
*Micrococcus* spp	12	5.1
*P.aeruginosa*	4	1.7
*Viridans Streptococcus*	2	0.9
*E. faecalis*	2	0.9
*Enterococcus* spp	1	0.4
*Aspergillus* spp	37	15.8
*Rhodotorula* spp	4	1.7
*Rhizopus* spp	1	0.4
Total N (%)	234	100

### Distribution of microbial pathogens across different operating theatres of Mbale regional hospital-eastern Uganda

Table 4 Most of the microbial pathogens were isolated from Ophthalmology theatre, 38.9% (91/234). The major pathogens in this theatre were *pseudomonas* species, 22% (20/91) and *Bacillus spp*,18.7% (17/91). Gynaecology theatre was mostly contaminated with Pseudomonas spp. as high as 25.8% (16/62). General (Public) OT was mainly contaminated by Pseudomonas spp., 19.6% (9/46) and coagulase negative staphylococcus (CONS), 15.2% (7/46).

**Table 4:** General distribution of microbial pathogens in the different operating theatres of Mbale Regional Hospital, Eastern Uganda

	Theatre				
	Ophthalmology (*n* = 91)	Gynaecology (*n* = 62)	General-private (*n* = 35)	General-Public (*n* = 46)	*N* = 234
Organism n (%)					
*Pseudomonas spp*	20 (35.7)	16 (28)	11 (19.6)	9 (16.1)	
*Bacillus spp*	17 (41.5)	12 (29.3)	6 (14.6)	6 (14.6)	
*CoNS*	13 (54.2)	4 (16.7)	0 (0)	7 (29.2)	
*Enterobacter aerogens*	9 (45)	4 (20)	2 (10)	5 (25)	
*S. aureus*	4 (22.2)	8 (44.4)	3 (16.7)	3 (16.7)	
*E. coli*	6 (50)	2 (16.7)	2 (16.7)	2 (16.7)	
*Micrococcus spp*	5 (41.7)	5 (41.7)	1 (8.3)	1 (8.3)	
*P. aeruginosa*	3 (75)	0 (0)	0 (0)	1 (25)	
*Viridans Streptococcus*	0 (0)	0 (0)	2 (100)	0 (0)	
*E. faecalis*	0 (0)	0 (0)	1 (50)	1 (50)	
*Enterococcus spp*	1 (100)	0 (0)	0 (0)	0 (0)	
*Aspergillus spp*	12 (32.4)	8 (21.6)	7 (18.9)	10 (27)	
*Rhodotorula spp*	1 (25)	2 (50)	0 (0)	1 (25)	
*Rhizopus spp*	0 (0)	1 (100)	0 (0)	0 (0)	
Total N (%)	91 (36.8)	63 (25.5)	40 (16.2)	53 (21.5)	234 (100.0%)

### Distribution of microbial pathogens over different surfaces in operating theatres of Mbale regional hospital-eastern Uganda

Table 5 Operating bed, instrument trolley and door handles had the highest number of pathogens. Instrument trolley was mostly contaminated with *Pseudomonas* spp., 28.6% (8/28) and 77.8% (7/9) of *E. coli* contaminated the operating bed. 100% of *Staphylococcus aureus* were isolated from door handles. *Streptococcus viridae* was exclusively isolated from the operating bed in the general ward (private).

**Table 5:** Distribution of common microbial pathogens over different surfaces in operating theatres of Mbale Regional Hospital, Eastern Uganda

Surface	Organism n (%)				
	*Pseudomonas spp*	*Bacillus spp*	*E. coli*	*S. aureus*	*Streptoccus viridae*
Sink	3 (10.7)	0 (0.0)	0 (0.0)	0 (0.0)	0 (0.0)
Instrument trolley	8^a^(28.6)	1(11.1)	1(11.1)	0 (0.0)	0 (0.0)
Sterilizing drum	0(0.0)	0(0.0)	0(0.0)	0 (0.0)	0 (0.0)
Operating bed	4(14.3)	1(11.1)	7(77.8)	0 (0.0)	1 (50.0)
Wall	1(3.6)	0(0.0)	0(0.0)	0 (0.0)	0 (0.0)
Door handles	1(3.6)	1(11.1)	0(0.0)	2(100.0)	1(50.0)
IV fluid stand	2(7.1)	2(22.2)	0(0.0)	0 (0.0)	0 (0.0)
Oxygen mask	1(3.6)	0(0.0)	0(0.0)	0 (0.0)	0 (0.0)
Scrubbing tank	2(7.1)	0(0.0)	0(0.0)	0 (0.0)	0 (0.0)
Floor	3(10.7)	2(22.2)	1(11.1)	0 (0.0)	0 (0.0)
window	3(10.7)	2(22.2)	0(0.0)	0 (0.0)	0 (0.0)
Total	*n* = 28	*n* = 9	*n* = 9	*n* = 2	*n* = 2

^a^3 isolates are *Pseudomonas aeruginosa*

### Distribution of microbial pathogens in the air and on surfaces in the operating theatres of Mbale regional referral hospital, eastern Uganda

Table 6 Generally, theatre air was more contaminated than the surfaces at 69.7% (163/234). *Staphylococcus aureus* and coagulase negative staphylococcus (CoNS) were predominant on settle plates (air) than surfaces (*p* = 0.02). Air was most contaminated with micrococcus compared to in-theatre surfaces (*p* = 0.03). Aspergillus spp., *Rhodotorula* spp. were contaminating air many times compared to the in-theatre surfaces (*p* = 0.01). Conversely Viridans streptococcus and Enterococcus spp. were contaminating surfaces more than theatre aerial space.

**Table 6:** Distribution of microbial pathogens in the air and on surfaces in the operating theatres of Mbale Regional Referral Hospital, Eastern Uganda

	Medium		
	Air (settle plate) [*n*^a^=163]	Surface (Swab) [*n*^a^=71]	*p*-value
Organism n (%)			
*Pseudomonas* spp	27 (48.2)	29 (51.8)	0.94
*Bacillus* spp	32 (78.0)	9 (22.0)	0.15
*CoNS*	23 (95.8)	1 (4.2)	0.02
*Enterobacter aerogens*	6 (30.0)	14 (70.0)	0.30
*S. aureus*	16 (88.9)	2 (1.1)	0.02
*E. coli*	3 (25.0)	9 (75.0)	0.19
*Micrococcus* spp	11 (91.7)	1 (8.3)	0.03
*P.aeruginosa*	1 (25.0)	3 (75.0)	0.19
*Viridans Streptococcus*	0 (0.0)	2 (100)	0.01
*E. faecalis*	2 (100)	0 (0.0)	0.01
*Enterococcus* spp	0 (0.0)	1 (100)	0.01
*Aspergillus* spp	37 (100)	0 (0.0)	0.01
*Rhodotorula* spp	4 (100)	0 (0.0)	0.01
*Rhizopus* spp	1 (100)	0 (0.0)	0.01

^a^number of isolates

### Antimicrobial sensitivity pattern of isolates from operating theatres of Mbale regional referral hospital, eastern Uganda

**Fig. 1:**
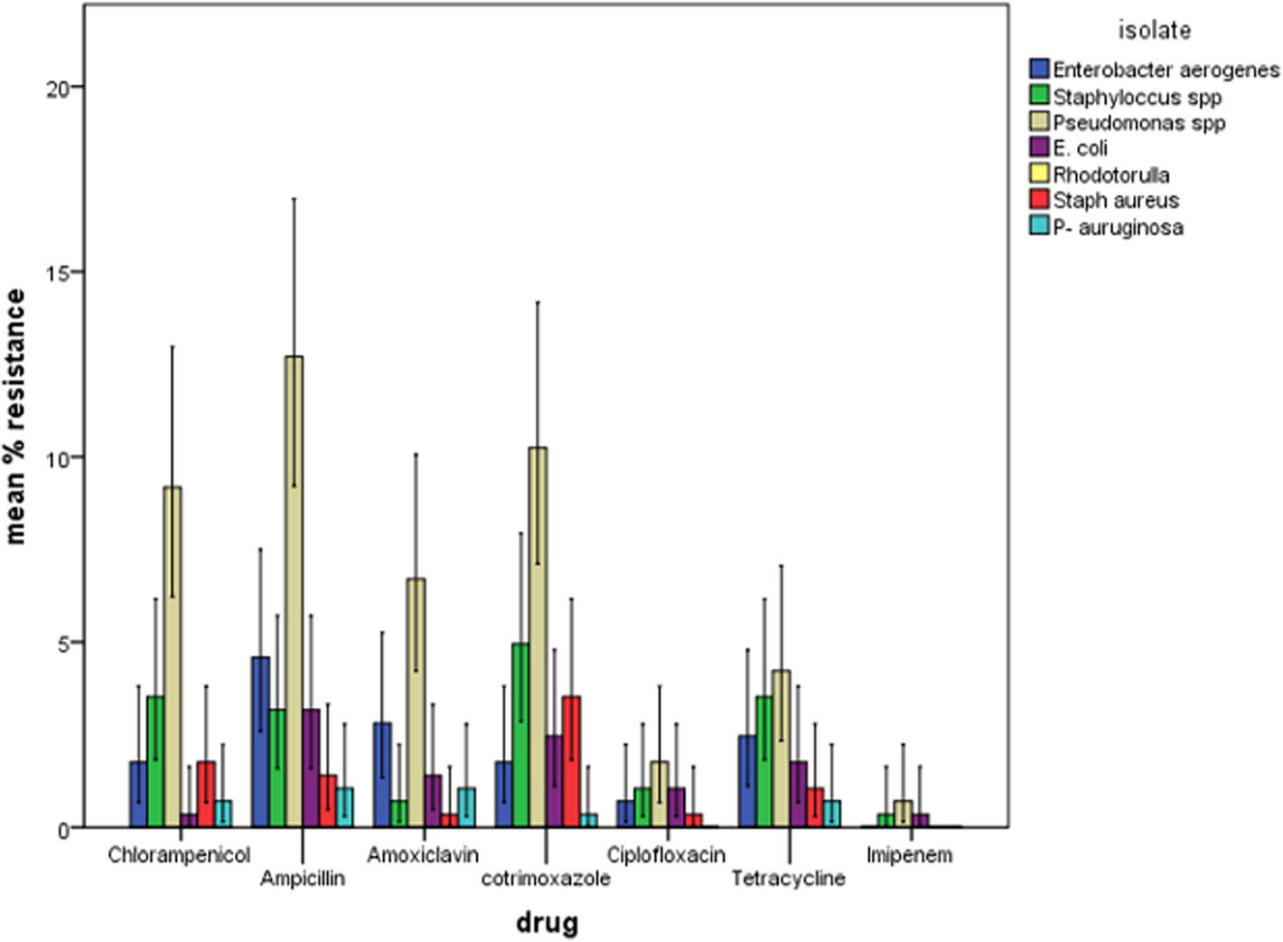
Resistance pattern of microbial pathogens in operating theatres of Mbale Regional Referral Hospital, Eastern Uganda

**Fig. 2:**
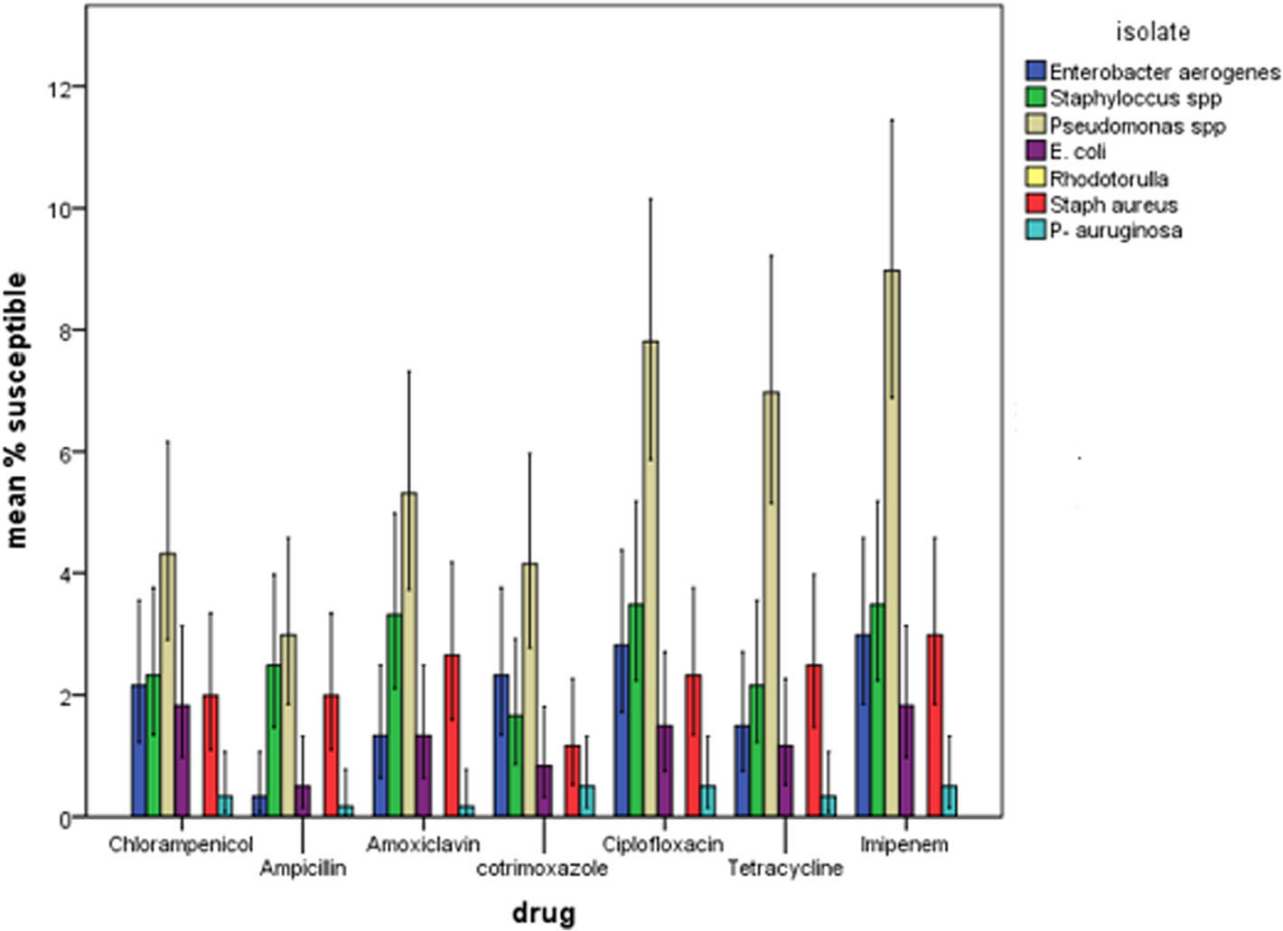
Susceptibility pattern of microbial pathogens in operating theatres of Mbale Regional Referral Hospital, Eastern Uganda

Figures 1 and 2 Most of the organisms were resistant to ampicillin, cotrimoxazole and chloramphenicol. *Pseudomonas* spp. was the most resistant bacteria followed by *Staphylococcus* spp. and *Enterobacter aerogenes*. Imipenem and ciprofloxacin were the antibiotics with the highest activity.

## Discussion

In this study, the mean colony counts in all the four operating theatres ranged between 44 and 70 cfu/dm^2^/h which is far above the acceptable limit of 5 cfu/dm^2^/h according to Fisher’s Index of Microbial air contamination [16]. *Pseudomonas* spp. (24%) and *Bacillus* spp. (18%) were the mostly isolated bacteria. Our findings are lower than those found in operating theatres of Edwin Cade Memorial Hospital in Ghana where bacillus comprised of 48.5% [17]. Bacillus is mostly associated with dusty environments which is common with Sub-Saharan Africa especially rural and semi-urban settings. Other micro-organisms isolated included *Coagulase Negative Staphylococcus, Enterobacter aerogens, S. aureus, E. coli, Micrococcus* spp.*, P.aeruginosa, Viridans Streptococcus, E. faecalis, Enterococcus* spp.*, Aspergillus* spp.*, Rhodotorula* spp. *and Rhizopus* spp. These findings are similar to those obtained from post-operative wound swabs in Mbale Regional referral hospital [18]. This suggests that environmental contamination of operating theatres with microbes may be the source of complicating post-operative wound sepsis [5].

Ophthalmology operating theatre was the most contaminated (39%) followed by Gynaecology operating theatre (27%). Our findings are different from those obtained by Kiranmai et al. who found reduced contamination (within acceptable limits) of Ophthalmology and Gynaecological operating theatres of Mediciti Medical College, Ghanpar, Hyderebad Telengana India [19]. The discrepancy is due to varying frequencies and intensity of fumigation of these operating theatres. In Mbale regional referral hospital, fumigation is not done daily in the Ophthalmology theatre as it is the case in other operating theatres which are presumed to be busy. Pseudomonas spp. was the most common bacteria isolated in Ophthalmology and Gynaecology operating theatres. Our findings are similar to those found by Ensayef et al. in Al Iman Ali hospital, Baghdad [2]. Also P. aeruginosa was the most common in the Gynaecology theatre. The source of this organism could be antiseptic solution or breast-fed babies and *P. aeruginosa* can be a contributor of outbreaks in operating theatres [20, 21].

In Uganda, regional referral hospitals and other public health facilities are not well facilitated with hospital supplies and reagents and chances of keeping prepared antiseptic solution on bench for long is most likely. Long storage of prepared antiseptic solutions facilitates contamination by Pseudomonas spp. leading to wide spread contamination in the theatre environment.

It is interesting that general theatre was less contaminated than Gynaecological theatre, this is similar to reports from Mbarara Regional Referral Hospital where *Staphylococcus aureus, Bacillus* spp.*, E. coli, Pseudomonas* spp.*, Aspergillus* spp.*, Rhizopus* spp.*, Micrococcus* spp. were the dominant isolates from gynaecology operating theatres [22]. The high level of contamination in the Gynecology theatre could be attributed to huge gynaecological and obstetric patient in flow especially in semi-urban referral settings. Nosocomial infections in this setting necessarily require attention as they have been found to aggravate maternal and neonatal mortality especially in a rural setting [23].

Coagulase Negative Staphylcoccus (CONS) was most common (29%) in General operating theatre (Public) compared to other operating theatres. Our findings are similar to those found by Ensayef et al. in Baghdad, Iraq in the general ward [2]. The possible source of CONS is endogenously normal skin flora and exogenously from surgical staff [24].

Operating bed [in the gynaecological theatre] was heavily contaminated with E. coli. This suggests fecal contamination of the operating bed. Our findings are of great public health importance in view of fecal contamination and nosocomial infection transmission respectively. E. coli has been found to complicate wound sepsis resulting into poor patient prognosis [25, 26].

However, the door handles were contaminated with *Staphylococcus aureus* (100%). These findings are comparable to those from a study conducted in Yamaguchi University Hospital, Ube Japan where 27% of the door handles were contaminated with *Staphylococcus aureus* [27]. This implies that door handles can be a source of nosocomial infection in operating theatres leading to complicated wound infections.

Aerial space (Settle plates) were contaminated with coagulase negative staphylococcus. This suggests in adequacy in ventilation system of the operating theatres as well as cleaning procedures [28–30]. These findings are comparable to findings obtained at Jimma Ethiopia [31]. Coagulase negative *Staphylococcus* in our study setting could have come from skin of patients or staff as well as from outside environment owing to poor ventilation system of the theatres.

Many of the microbial pathogens isolated presented a considerable level of resistance to antimicrobial agents. This is so due to frequent abuse of antibiotic use especially in developing countries [29] . *pseudomonas* spp. was considerably resistant to chloramphenicol (53.6%) and cotrimoxazole (57.1%). These findings are similar to those found in USA where *Pseudomonas* has shown considerable resistance to B-lactam and cepharosporin antibiotics [32]. The Multi-drug resistance of *Pseudomonas* spp. is mainly due to the bacteria’s ability to produce inducible Amp C B-lactamases [33] that can easily be induced by B-lactam possessing antibiotics and thereafter get hydrolysed. *Pseudomonas auruginosa* is capable of copiously manufacturing a porin (OPrF) that forms a large outer membrane pore for impermeability to the broad spectrum antibiotic [34]. Gram negative isolates showed the highest resistance to ampicillin. Escherichia coli and Enterobacter aerogenes presented 75% and 83.3% resistance to ampicillin respectively. Our findings can be explained by intrinsic resistance of E. coli and Enterobacter aerogenes by way of possessing cascades that guard cell membrane permeability as well as producing Amp C B-lacatamase [35]. Enterobacter aerogenes has been associated with nosocomial infections in Europe [36–41].

Imipenem was the agent to which all bacteria exhibited highest sensitivity. Imipenem could be a drug of use in our setting, however, prescription of imipenem should be done carefully. Carmel et al. [42] found out there is 2-fold increased risk of drug resistance to most antibiotics when imipenem is used as compared to when ciprofloxacin, ceftazidime or even piperacillin is used.

The study had weaknesses: During the study period, the surgical site infections were not able to be captured as well as the associated pathogens since it is not a routine diagnostic procedure to perform culture and sensitivity in this hospital. Additionally, we were unable to sample Casualty Operating Theatre and so cannot ascertain the microbial contamination in this theatre. The study ‘s strengths: According to our knowledge, this is the first study to include both aerial and surface contamination of operating theatres of Eastern Uganda. In practice, theatre workers and surgeons ensure aseptic surgical techniques despite the non-excellent structure of the theatres in rural Eastern Uganda.

## Conclusions

Microbial contamination of operating theatres in Mbale regional hospital exceed acceptable limits. *Pseudomonas*, *Bacillus* and *Aspergillus* spp. form the majority of the contaminants. Antibiotic resistance to chloramphenicol and cotrimoxazole was noted. Imipenem and ciprofloxacin showed highest bacterial sensitivity.

Thorough and regular disinfection of surgical theatres especially Ophthalmology and Gynaecology are necessary in referral settings. Recommendation of sulfonamides and aminoglycosides as antibiotic therapy in semi-urban referral settings should be done with care due to envisaged resistance. Use of imipenem should not be recommended as treatment of choice as it facilitates resistance to several antibiotics. Additionally, more studies are recommended to study relationship between surgical site infection, especially for emergency surgeries, and microbial contamination of various operating theatres in Mbale Regional referral hospital and other referral settings of rural Uganda.

## Additional files

Additional file 1: **Data S1.** Microbial pathogen contamination of operating theatres of Mbale Regional Referral Hospital, Eastern Uganda. (CSV 3 kb)

Additional file 2: **Data S2.** Susceptibility pattern of microbial pathogens isolated from operating theatres of Mbale Regional Referral Hospital, Eastern Uganda**.** Indicating value labels for data 1 and data 2. (CSV 24 kb)

Additional file 3: Data description (DOCX 13 kb)

## Abbreviations

ATCC: American Typed Culture Collection
Cfu/dm^2^/h: Colony forming units per square decimeter per hour
CLSI: Clinical and laboratory standard institute
CONS: Coagulase negative staphylococcus
DE: Design effect
F: Fisher’s ratio
HAIs: Hospital acquired infections
ISO: International Organization for Standardization
MUST: Mbarara University of Science and Technology
OPrF: Monomeric outer membrane protein for Pseudomonas
OT: Operating theatre
RRH: Regional referral hospital
SPP(s): Specie(s)
SPSS: Statistical package for social scientists
WHO: World Health Organization

## References

[1] Okon KO (2012). Bacterial contamination of operating theatre and other specialized care unit in a tertiary hospital in northeastern Nigeria. Afr J Microbiol Res.

[2] Ensayef S, Al-Shalchi S, and S M, Microbial contamination in the operating theatre: a study in a hospital in Baghdad Eastern Mediterranean Health Journal. 2009;15(1):219–23. 19469446

[3] Edmiston CE (2005). Molecular epidemiology contamination of microbial in the operating room environment: is there a risk for infection?. Surgery.

[4] J N (2006). Microbial flora on operating room telephones. AORON.

[5] Laham NAA (2012). Prevalence of bacterial contamination in general operating theaters in selected hospitals in the Gaza strip, Palestine. Journal of Infection and Public Health.

[6] Reddy BR (2012). Management of culture-negative surgical site infections. J Med Allied Sci.

[7] Akhtar N (2010). Hospital acquired infections in a medical intensive care unit. Coll Physicians Surg Pak.

[8] Tesfaye T, Berhe Y, Gebreselassie K. Microbial contamination of operating Theatre at Ayder Referral Hospital, Northern Ethiopia. International Journal of Pharma Sciences and Research (IJPSR). 2015:6 (10).

[9] Al Laham NA (2012). Distribution and Antimicrobial Resistance Pattern of Bacteria Isolated from Operation Theaters at Gaza Strip. Journal of Al Azhar University-Gaza (Natural Sciences).

[10] Napoli C, Marcotrigiano V, Montagna MT (2012). Air sampling procedures to evaluate microbial contamination: a comparison between active and passive methods in operating theatres. BMC Public Health.

[11] Nasser AMa (2013). Assessment of surgical iite infections from signs, symptoms of the wound and associated factors in public hospitals of Hodeidah city. Yemen IntJAppSciTech.

[12] WJ M, RL N (2001). Recognition, prevention, surveillance and management of SSI. Clin Infect Dis.

[13] Lubega A, Joel B, Justina Lucy N (2017). Incidence and etiology of surgical site infections among emergency postoperative patients in Mbarara regional referral hospital. South Western Uganda Surg Res Pract.

[14] Kitembo SK, Chugulu SG (2013). Incidence of surgical site infections and microbial pattern at kilimanjaro christian medical centre. Annals of African Surgery.

[15] CLSI (2008). Abbreviated Identification of bacteria and Yeast; Approved guideline.

[16] Saha R, Agarawal S, AM K (2017). Air sampling procedures to evaluate microbial contamination: a comparison between active and passive methods at high-risk areas in a tertiary Care Hospital of Delhi. J Patient Saf Infect Control.

[17] Feglo P, Afriyie-Asante A (2014). Environmental impact on postoperative wound infections in a privately owned hospital in Ghana. Afr J Microbiol Res.

[18] George M (2015). Prevalence of bacterial pathogens and their antibiotic susceptibility patterns among patients with post-operative wound infections at Mbale Hospital in SBLS-CoVAB.

[19] Kiranmai S, Madhavi K. Microbiological surveillance of operation theatres, intensive care units and labor room of a teaching hospital in Telangana, India. International Journal of Research in Medical Sciences. 2016:5256–60.

[20] Bellido F, Hancock R, Campa M, Bendinelli M, F H (1993). Susceptibility and resistance of P. aeruginosa to antimicrobial agents, in Pseudomona aeruginosa as an opportunistic pathogen.

[21] C, P (2003). An outbreak of carbapenemresistant Pseudomona aeruginosa in a urology ward. Clin Microbiol Infect.

[22] Ampaire L, Okonye J (2014). Assessment of the current level of sterility in surgical and Gynaecology theatres at Mbarara Regional Referral Hospital. Kigali, in The fourth scientific conference of Rwanda Association of Biomedical Technologists.

[23] Halder A, Vijayselvi R, Jose R (2015). Changing perspectives of infectious causes of maternal mortality. J Turk Ger Gynecol Assoc.

[24] KL G (1995). Infection after total hip arthroplasty. Bone and joint surgery.

[25] Moremi N (2017). Surveillance of surgical site infections by Pseudomonas aeruginosa and strain characterization in Tanzanian hospitals does not provide proof for a role of hospital water plumbing systems in transmission. Antimicrob Resist Infect Control.

[26] Petkovšek Ž (2009). Virulence potential of Escherichia coli isolates from skin and soft tissue infections. ▿ Clinical Microbiology.

[27] Oie S, Hosokawa I, Kamiya A (2002). Contamination of room door handles by methicillin-sensitive/methicillin-resistant Staphylococcus aureus. Hospital Infection.

[28] Fleischer M (2005). Microbiological control of airborne contamination in Hopsital. Indoor built environ.

[29] Ahmed Abdel Gawad Elmasry, et al., Pattern of antibiotic abuse – a population based study in Cairo. Egyptian Journal of Chest Diseases and Tuberculosis, 2013. 189–195: p. 189–195.

[30] Holton J, GL R (1993). Commissioning operating theatres. J Hosp Infect Immun.

[31] Genet C, Kibru G, Tsegaye W (2011). Indoor air bacterial load and antibiotic susceptibility pattern of isolates in operating rooms and surgical wards at Jimma University specialized hospital, Southwest Ethiopia. Ethiop J Health Sci.

[32] Henwood CJ, Livermore DM, and James D. Warner M, The Pseudomonas Study Group. Antimicrobial susceptibility of Pseudomonas aeruginosa: results of a UK survey and evaluation of the British Society for Antimicrobial Chemotherapy disc susceptibility test,. J Antimicrob Chemother 2001. 47: p. 789–799). 10.1093/jac/47.6.789 11389111

[33] DM L (1995). β -lactamases in laboratory and clinical resistance. Clin Microbiol Rev.

[34] Benz R, RE H (1981). Properties of the large ion-permeable pores formed from protein F of Pseudomonas aeruginosa in lipid bilayer membranes. Biochim Biophys Acta.

[35] Davin-Regli A, Pagès J-M (2015). Enterobacter aerogenes and Enterobacter cloacae; versatile bacterial pathogens confronting antibiotic treatment. Fron Microbiol.

[36] Allerberger F (1996). Epidemiology of infections due to multiresistant Enterobacter aerogenes in an university hospital. Eur J Clin Microbiol Infect Dis.

[37] Arpin C (1996). Epidemiological study of an outbreak due to multidrug-resistant Enterobacter aerogenes in a medical intensive care unit. J Clin Microbiol.

[38] Davin-Regli A (1996). Molecular epidemiology of Enterobacter aerogenes acquisition: one-year prospective study in two intensive care units. J Clin Microbiol.

[39] De Gheldre Y (1997). Molecular epidemiology of an outbreak of multidrug-resistant Enterobacter aerogenes infections and in vivo emergence of imipenem resistance. J Clin Microbiol.

[40] Georghiou PR (1995). Molecular epidemiology of infections due to Enterobacter aerogenes: identification of hospital outbreak-associated strains by molecular techniques. Clin Infect Dis.

[41] Grattard F (1995). Characterization of nosocomial strains of Enterobacter aerogenes by arbitrarily primed PCR analysis and ribotyping. Infect Control Hosp Epidemiol.

[42] Carmeli Y (1999). Emergence of antibiotic-resistant Pseudomonas aeruginosa: comparison of risks associated with different antipseudomonal agents. Antimicrob Agents Chemother.

