# Supplementary material for: Contamination of microbial pathogens and their antimicrobial pattern in operating theatres of peri-urban eastern Uganda: a cross-sectional study
Source: BMC Infect Dis. 2018 Sep 10;18:460. doi: 10.1186/s12879-018-3374-4 (PMC6131813; doi:10.1186/s12879-018-3374-4)
Supplement: Supplementary file 3 — Data description (DOCX 13 kb) [file 12879_2018_3374_MOESM3_ESM.docx]

**Data description**

| **Data 1** | |
| --- | --- |
| variable | Value & label |
| Theatre_ | 1=ophthalmology  2=gynaecology  3=general private  4=main OT (general public) |
| Sample type | 1=settle plate  2=surface |
| Spot_collect | 1=sink; 2=instrument trolley; 3=sterilization drum; 4=operating bed; 5=wall; 6=baby trolley; 7=door handle; 8=IV fluid stand; 9=air; 11=oxygen cylinder; 12=scrubbing tank; 13=floor; 14=window |
| isolate | 1=aspergillus spp; 2=enterobacter aerogenes; 4=bacillus spp; 5=staphylococcus spp; 6=pseudomonas spp; 7=micrococcus spp; 8=E. coli; 9=rhodotorula spp; 10=Rhizopus; 11=aspergillus fumigatus; 12=aspergillus flavus; 13=aspergillus niger; aspergillus tereus; 14=enterococcus; 21=enterococcus; 41= staphylococcus aureus; 61=P. auruginosa; 91=streptococcus viridae; 211=enterococcus feacalis |

| **Data 2** | |
| --- | --- |
| variable | Value & label |
| drug | 1=ceftriaxone; 2=gentamicin; 3=chloramphenicol; 4=ampicillin; 5=amoxicillin; 6=cotrimoxazole; 7=ciprofloxacin; 8=tetracycline; 9=imipenem |
| isolate | 1=aspergillus spp; 2=enterobacter aerogenes; 4=bacillus spp; 5=staphylococcus spp; 6=pseudomonas spp; 7=micrococcus spp; 8=E. coli; 9=rhodotorula spp; 10=Rhizopus; 11=aspergillus fumigatus; 12=aspergillus flavus; 13=aspergillus niger; aspergillus tereus; 14=enterococcus; 21=enterococcus; 41= staphylococcus aureus; 61=P.auruginosa; 91=streptococcus viridae; 211=enterococcus feacalis |
| susceptibility | 0=resistant  3=susceptible |
